# Defects and dopant properties of Li_3_V_2_(PO_4_)_3_

**DOI:** 10.1038/s41598-018-36398-w

**Published:** 2019-01-23

**Authors:** Navaratnarajah Kuganathan, Alexander Chroneos

**Affiliations:** 1 Department of Materials, Imperial College London, London, SW7 2AZ United Kingdom; 2 Faculty of Engineering, Environment and Computing, Coventry University, Priory Street, Coventry, CV1 5FB United Kingdom

## Abstract

Polyanion phosphate based Li_3_V_2_(PO_4_)_3_ material has attracted considerable attention as a novel cathode material for potential use in rechargeable lithium ion batteries. The defect chemistry and dopant properties of this material are studied using well-established atomistic scale simulation techniques. The most favourable intrinsic defect process is the Li Frenkel (0.45 eV/defect) ensuring the formation of Li vacancies required for Li diffusion via the vacancy mechanism. Long range lithium paths via the vacancy mechanism were constructed and it is confirmed that the lowest activation energy of migration (0.60 eV) path is three dimensional with curved trajectory. The second most stable defect energy process is calculated to be the anti-site defect, in which Li and V ions exchange their positions (0.91 eV/defect). Tetravalent dopants were considered on both V and P sites in order to form Li vacancies needed for Li diffusion and the Li interstitials to increase the capacity respectively. Doping by Zr on the V site and Si on the P site are calculated to be energetically favourable.

## Introduction

The increased need for electrical energy storage for static and mobile applications in conjunction with the demand for higher capacity, better safety, increased cycle performance, and durability made the solid-state lithium batteries technologically important^1,2^. The research is focusing mainly on the electrolyte and cathode trying to identify more efficient materials^3–17^. In particular, electrode materials require should satisfy safety requirements, be low cost, and critically have a higher energy density (i.e. large density of Li^+^ ions).

State of the art cathodes for solid-state lithium batteries include polyanion-type oxides, layered lithiated transition metal oxides and Mn-based spinels^18^. Monoclinic Li_3_V_2_(PO_4_)_3_ has gained the interest of the community as a cathode material for solid-state lithium batteries due to its low cost, safety, low environmental impact, appropriate cycling stability, and high theoretical capacity (197 mAhg^−1^)^18–25^. Fu *et al*.^26^ synthesized this material using mixed lithium precursors with particles high surface area leading to good electrochemical performance. Solid state^7^ Li NMR together with two-dimensional exchange study of Lithium was used to determine the temperature dependent Li hoping process to understand the Li dynamics on the microscopic scale suggesting that their methodology can be applied to the cycled materials and other lithium metal phosphates^27^. Lee and Park^22^ employed molecular dynamics simulation to calculate the vacancy migration energy of Li at different temperatures and confirmed the mobility of Li^+^ ions is anisotropic. Though these separate studies show different properties in this material, fundamental understating of this material is needed to optimize its performance.

Electrochemical behaviour of an electrode material is important to assess its applicabilty in batteries. This behavour can be studied theoretically by perfoming defect calculations and we note that such studies are absent in the literature. Atomistic modelling based on the classical pair potentials is a poweful method and can provide useful information about the defect processes, cation doping behavior and ion migration mechanism. This methodology has been applied to a variety of oxides including Li containing materials and excellent agreement in trends and energetics of defect processes was observed between calculation and the experiment^7,28,29^. Here, we extend our recent modelling of electrode materials^30–34^ where we examined the defects, ion diffusion and dopants. In the present study, we employ established atomistic modeling techniques to investigate the intrinsic defect chemistry, the impact of doping on the formation of lithium interstitials and lithium ion diffusion pathways in Li_3_V_2_(PO_4_)_3_. We consider the solution of a range of oxides in Li_3_V_2_(PO_4_)_3_ including aliovalent dopants (e.g. Si, Ge and Ti) and isovalent dopants (e.g. Al, Ga and Sc).

## Results and Discussion

### Li_3_V_2_(PO_4_)_3_ structure

**Figure 1:**
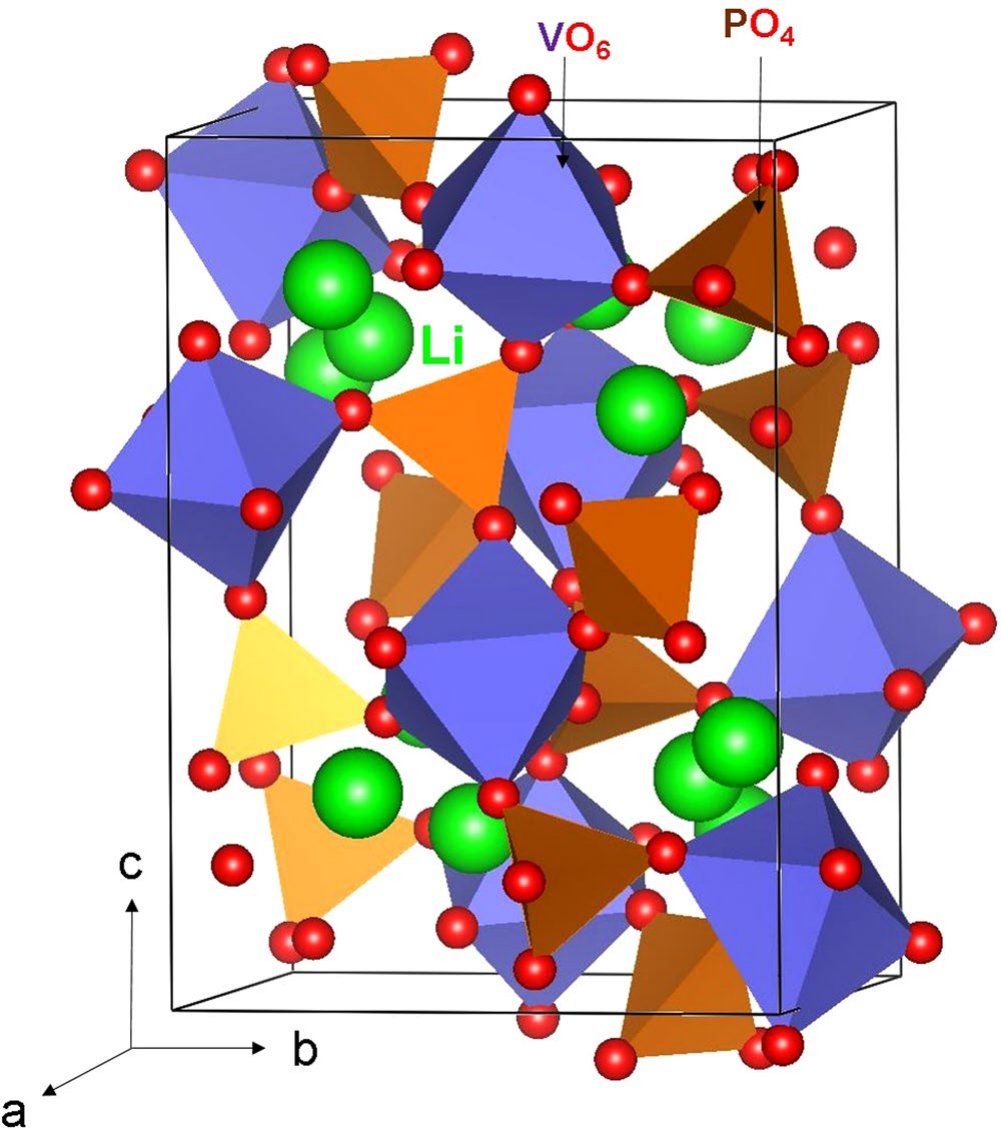
Crystal structure of Li_3_V_2_(PO_4_)_3_ (space group (P2_1_/*n*).

The crystal structure of Li_3_V_2_(PO_4_)_3_ exhibits a monoclinic crystallographic structure with space group *P*2_1_/*n* (lattice parameters a = 8.5978 Å, b = 8.5933 Å, c = 12.0327 Å, α = 90.0°, β = 90.496° and γ = 90.0°) as reported by Fu *et al*.^26^ Fig. 1 shows this structure and the chemical environment of P (forming tetrahedral unit with four O atoms) and V (forming octahedral unit with six O atoms). The starting point for the present study was to reproduce the experimentally observed monoclinic crystal structure to enable an assessment of the quality and efficacy of the classical pair potentials (refer to Table S1 in the supplementary information for the potentials parameters used and method section for the detailed description of the methodology) used here. The calculated equilibrium lattice constants (refer to Table 1) are in excellent agreement with experiment.

**Table 1:** Calculated and Experimental Structural Parameters for Monoclinic (P2_1_/*n*) Li_3_V_2_(PO_4_)_3_.

Parameter	Calc	Expt^13^	|∆|(%)
a (Å)	8.431424	8.597800	1.94
b (Å)	8.548920	8.593300	0.52
c (Å)	12.076179	12.032700	0.36
α (°)	90.0000	90.0000	0.00
β (°)	90.070	90.496	0.47
γ (°)	90.0000	90.0000	0.00

### Intrinsic defect processes

To understand the electrochemical behavior of an electrode material, intrinsic defect processes are crucial. A series of isolated point defect (vacancy and interstitial) energies were calculated, which were combined to determine the formation energies for Frenkel and Schottky-type defects in Li_3_V_2_(PO_4_)_3_. The following equations represent the reactions involving these defects as written using Kröger-Vink notation^35^.

1 $${\rm{Li}}\,{\rm{Frenkel}}:{{\rm{Li}}}_{{\rm{Li}}}^{{\rm{X}}}\to {V{\prime} }_{{\rm{Li}}}+{{\rm{Li}}}_{{\rm{i}}}^{\bullet }$$

2 $${\rm{O}}\,{\rm{Frenkel}}:{{\rm{O}}}_{{\rm{O}}}^{{\rm{X}}}\to {V}_{{\rm{O}}}^{\bullet \bullet }+{{\rm{O}}}_{{\rm{i}}}^{{\prime\prime} }$$

3 $${\rm{V}}\,{\rm{Frenkel}}:{V}_{{\rm{V}}}^{{\rm{X}}}\to {V\prime\prime\prime }_{{\rm{V}}}+{{\rm{V}}}_{{\rm{i}}}\bullet \bullet \bullet $$

4 $${\rm{P}}\,{\rm{Frenkel}}:{{\rm{P}}}_{{\rm{P}}}^{{\rm{X}}}\to {V}_{{\rm{P}}}^{{\prime} {\prime} {\prime} {\prime} {\prime} }+{\,{\rm{P}}}_{{\rm{i}}}^{\bullet \bullet \bullet \bullet \bullet }$$

5 $$\begin{array}{c}{\rm{Schottky}}:3{{\rm{Li}}}_{\mathrm{Li}\,}^{{\rm{X}}}+2{{\rm{V}}}_{{\rm{V}}}^{{\rm{X}}\,}+3{{\rm{P}}}_{{\rm{P}}}^{{\rm{X}}\,}+12{{\rm{O}}}_{{\rm{O}}}^{{\rm{X}}}\to 3{V{\prime} }_{{\rm{Li}}}+2{{\rm{V}}}_{{\rm{v}}}^{{\prime} {\prime} {\prime} }+3{V}_{{\rm{P}}}^{{\prime} {\prime} {\prime} {\prime} {\prime} }\\ \,\,\,\,+\,12{V}_{{\rm{O}}}^{\bullet \bullet }+{{\rm{Li}}}_{3}{{\rm{V}}}_{2}{({{\rm{PO}}}_{4})}_{3}\end{array}$$

6 $${{\rm{Li}}}_{2}{\rm{O}}\,{\rm{Schottky}}:2{{\rm{Li}}}_{{\rm{Li}}}^{{\rm{X}}}+{{\rm{O}}}_{{\rm{O}}}^{{\rm{ > X}}\,}\to 2{V{\prime} }_{{\rm{Li}}}+{V}_{{\rm{O}}}^{\bullet \bullet }+{{\rm{Li}}}_{2}{\rm{O}}$$

7 $${\rm{Li}}/{\rm{V}}\,\mathrm{antisite}\,({\rm{isolated}}):{{\rm{Li}}}_{{\rm{Li}}}^{{\rm{X}}}+{V}_{{\rm{V}}}^{{\rm{X}}\,}\to {{\rm{Li}}}_{{\rm{V}}}^{{\prime\prime} }+{{\rm{V}}}_{{\rm{Li}}}^{\bullet \bullet }$$

8 $${\rm{Li}}/{\rm{V}}\,\mathrm{antisite}\,({\rm{cluster}}):{{\rm{Li}}}_{{\rm{Li}}}^{{\rm{X}}}+{{\rm{V}}}_{{\rm{V}}}^{{\rm{X}}}\to {\{{{\rm{Li}}}_{{\rm{V}}}^{{\prime\prime} }:{{\rm{V}}}_{{\rm{Li}}}^{\bullet \bullet }\}}^{{\rm{X}}}$$

**Figure 2:**
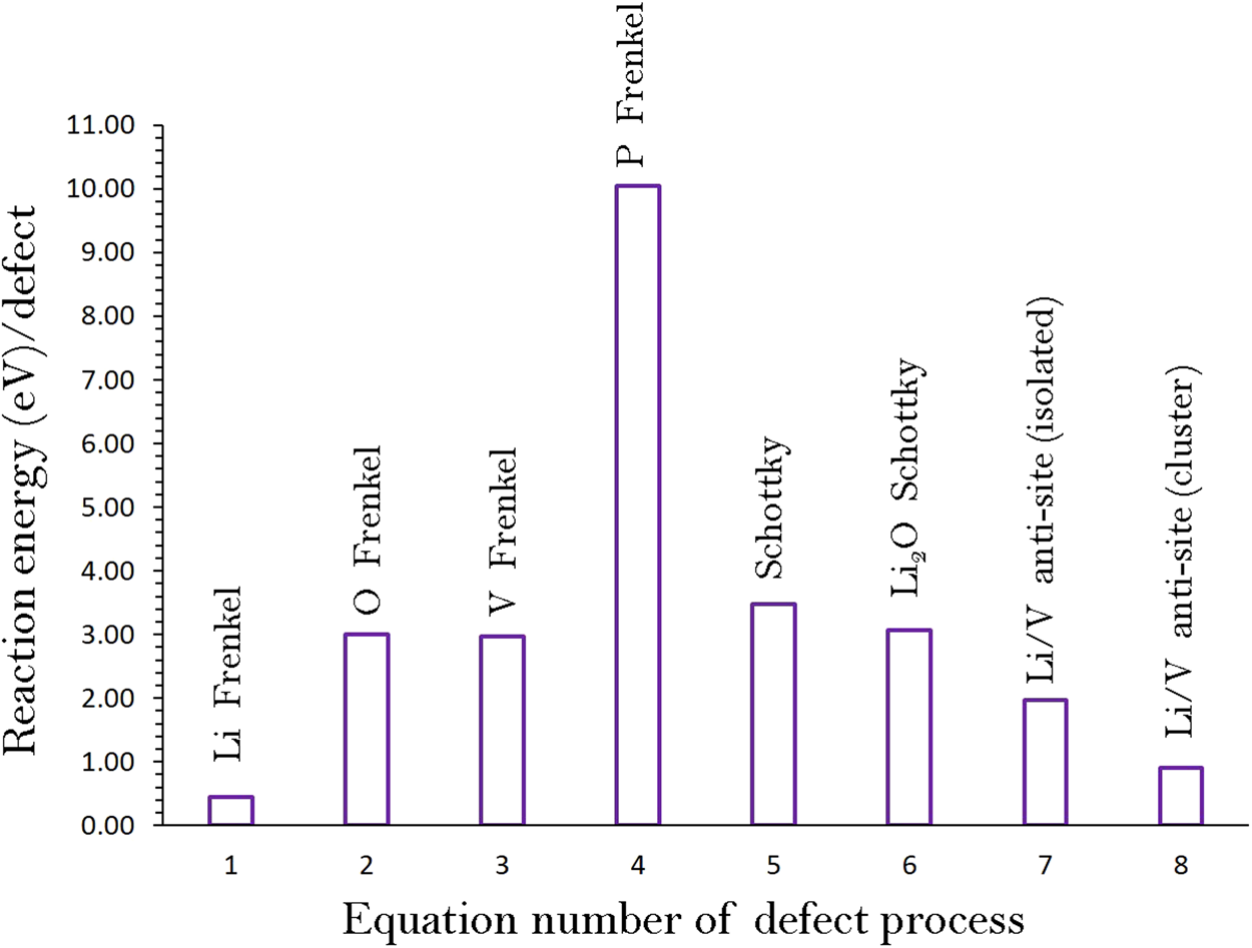
Energetics of intrinsic defect process in monoclinic Li_3_V_2_(PO_4_)_3_.

The reaction energies for these intrinsic defect processes are reported in Fig. 2 and Table S2. The most favourable intrinsic disorder is Li Frenkel and the formation of other Frenkel and Schottky defects is less energetically favourable. The second most favourable defect process is calculated to be Li-V anti-site. This indicates that there will be a small percentage of Li on V sites $({{\rm{L}}}_{{\rm{V}}}^{{\prime\prime} })$ and P on Li sites $({{\rm{V}}}_{{\rm{Li}}}^{\cdot \cdot })$ particularly at higher temperatures. In the relaxed configuration, there is insignificant changes observed in the cation-oxygen bond distances. Antisite defects have been observed experimentally and theoretically in a variety of Li ion cathode battery materials^30–33,36–41^. In the experimental study of as-prepared Li_2_MnSiO_4_, a small amount of Li-Mn anti-site defect was observed^41^. During cycling of Li_2_FeSiO_4_, Nyten *et al*.^36^ observed structural rearrangement in the crystal structure responsible for the Li-Fe anti-site. The difference between the isolated and cluster defect energies is calculated to be ‒1.06 eV suggesting that the anti-site cluster is stable compared tom its isolated form. The formation enthalpy of Li_2_O via the Li_2_O Schottky-like reaction (relation 6) is a processes that requires an energy of 3.07 eV per defect (refer to Table S2, supplementary information). This is a process that can lead to further $\,{V{\prime} }_{{\rm{Li}}}$ and ${V}_{O}^{\bullet \bullet }$, however, at elevated temperatures. The trend calculated for Li and V Frenkel and Li-V antisite defects is in agreement with the theoretical calculations performed by Lee *et al*.^22^, though there is a difference in defect energetics. Such difference is dependent on the choice of classical pair potentials used.

### Lithium ion-diffusion

**Figure 3:**
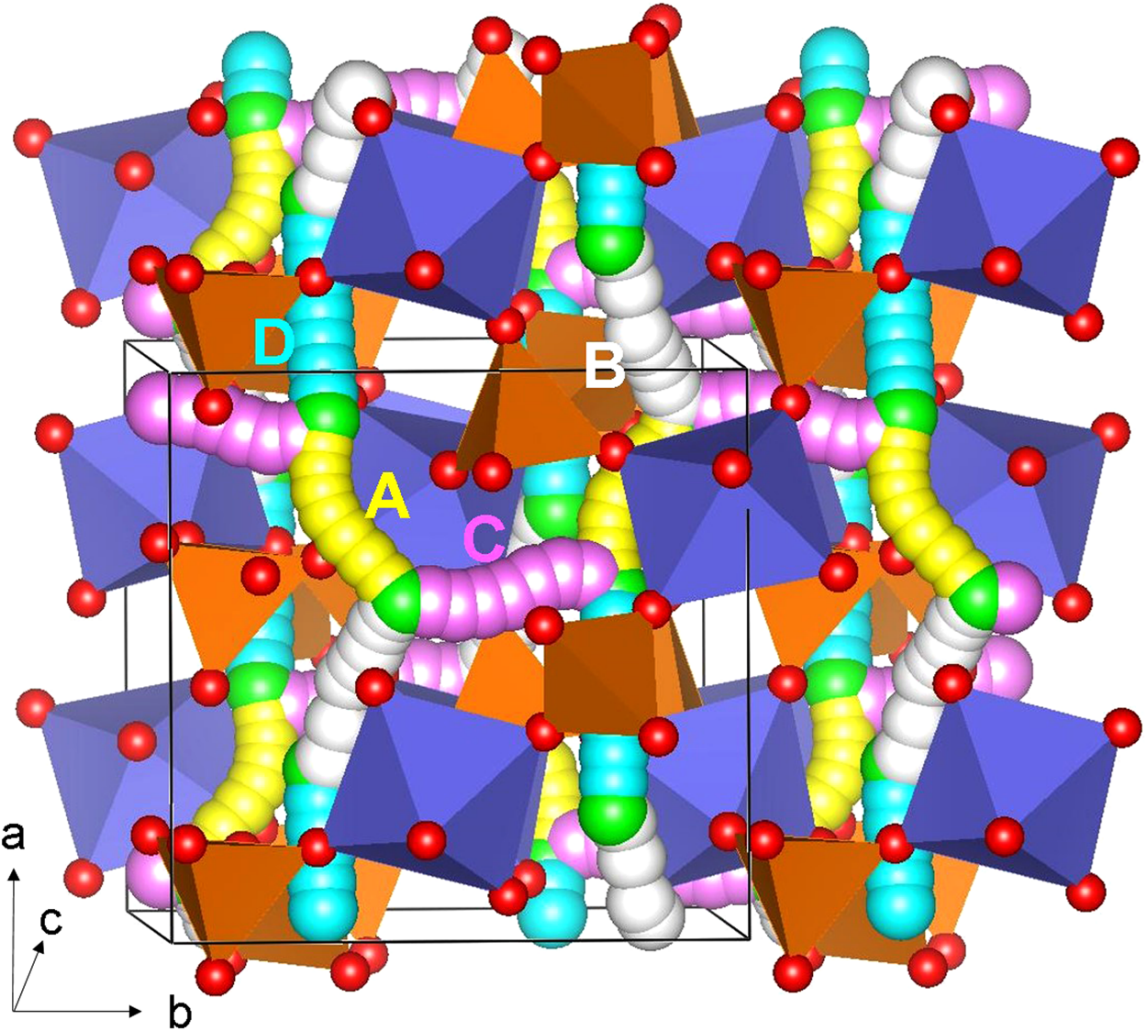
Possible long range sodium vacancy migration paths considered. Local Li migration paths are shown in blue, yellow, white and light purple atoms. PO_4_ and VO_6_ units are shown brown and violet colors respectively.

**Figure 4:**
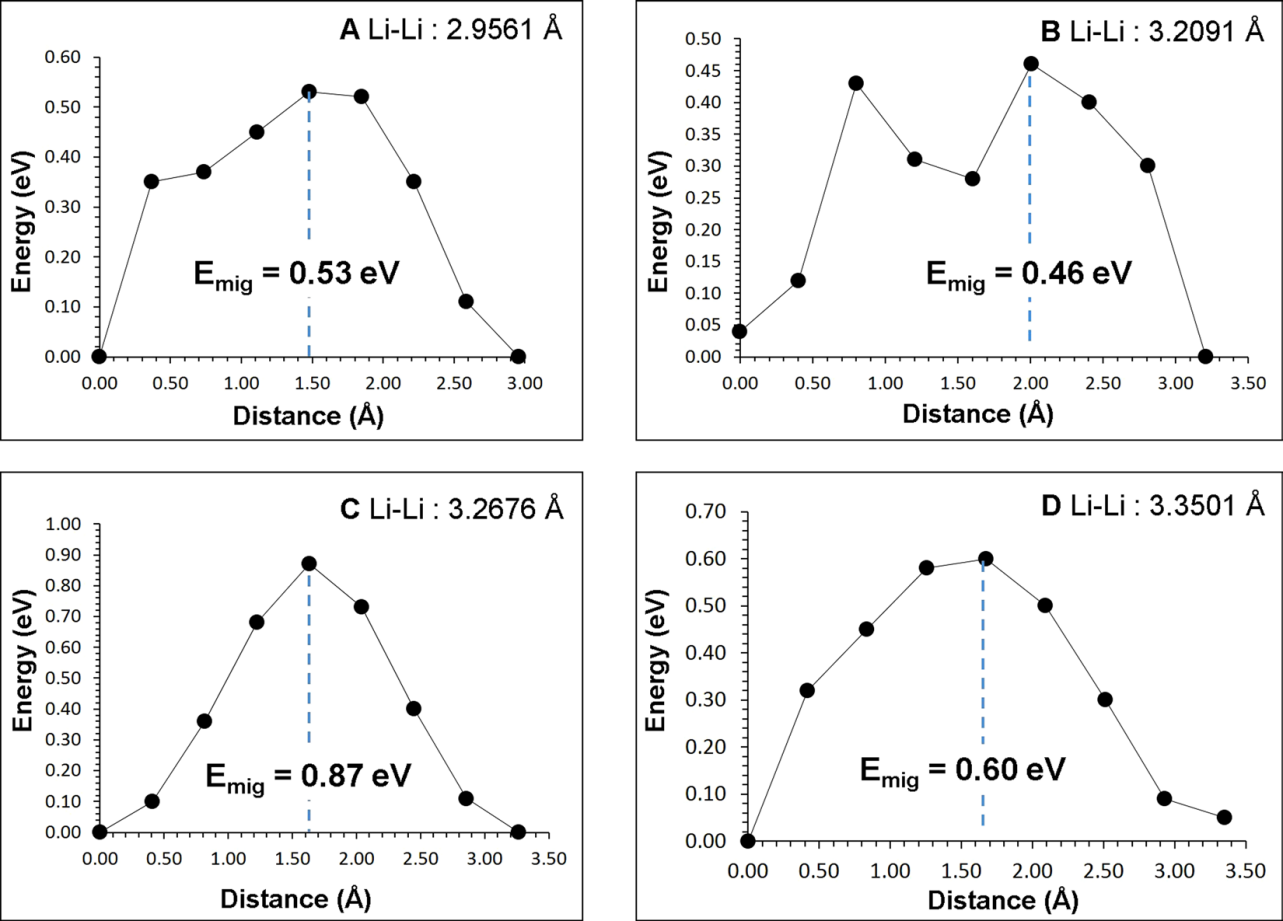
Four different energy profiles (as shown in Fig. 3) of Li vacancy hopping between two adjacent Li sites in Li_3_V_2_(PO_4_)_3_.

As the intrinsic lithium ion diffusion of Li_3_V_2_(PO_4_)_3_ material is of crucial importance when assessing its use as a possible high-rate cathode material in lithium batteries, we used the present static atomistic simulation to examine various possible diffusion paths responsible for Li ion conduction, which are often difficult to explore on the atomic scale by experiment alone. For the Li vacancy migration, we identified four lower activation energy local hops (A, B, C and D) and constructed long range paths connecting local Li hops (refer to Fig. 3). There are many long range three dimensional paths present. The lowest overall migration energy (0.60 eV) was calculated for the D → A → B → A path. Other possible long range paths were considered. However, the overall activation energy was calculated to be 0.87 eV due to the local hop C involved in the long range path. Here the activation energy of migration is defined as the position of the highest potential energy along the migration path. Migration energies are reported in Table 2 together with the Li-Li separation, whereas energy profile diagrams are shown in Fig. 4. Lee *et al*.^22^ calculated the one dimensional lithium ion diffusion mechanism in Li_3_V_2_(PO_4_)_3_ and their values deviates with our study. This is because in the present study we calculated three dimensional Li migation paths, which are the lowest energy migration paths (refer to Fig. 3 for detailed migration path). Migration paths calculated in this study exhibit curved trajecteries while in other studies, the paths are linear. Cahill *et al*.^27^ performed Li NMR measurements to estimate the activation energy for Li ion migration. The reported range of activation energies (0.73–0.83 eV) agrees reasonably with our calculated vlaues of 0.46–0.87 eV.

**Table 2:** Calculated Li-Li separations and activation energies for the sodium ion migration between two adjacent Li sites refer to Fig. 3.

Migration path	Li-Li separation (Å)	Activation energy (eV)
A	2.9561	0.53
B	3.2091	0.46
C	3.2676	0.87
D	3.3501	0.60

### Tetravalent doping

The Li Frenkel is calculated to be only 0.45 eV/defect; however, an increase in the concentration of Li will further increase the applicability of Li_3_V_2_(PO_4_)_3_ as a cathode material for rechargeable lithium batteries. A way to increase the content of intrinsic defects in oxides is by the solution of aliovalent dopants as it was previously demonstrated in CeO_2_ (for example ref.^42^ and references therein). Here we considered the solution of *RO*_2_ (*R* = Ce, Zr, Ti, Si and Ge) via the following process (in Kröger-Vink notation):

9 $$2{{\rm{RO}}}_{2}+2{{\rm{V}}}_{{\rm{V}}}^{{\rm{X}}}+2{{\rm{Li}}}_{{\rm{Li}}}^{{\rm{X}}}\to 2{{\rm{R}}}_{{\rm{V}}}^{\bullet }+2{{V}}_{{\rm{Li}}}^{{\prime} }+{{\rm{V}}}_{2}{{\rm{O}}}_{3}+{{\rm{Li}}}_{2}{\rm{O}}\,$$

**Figure 5:**
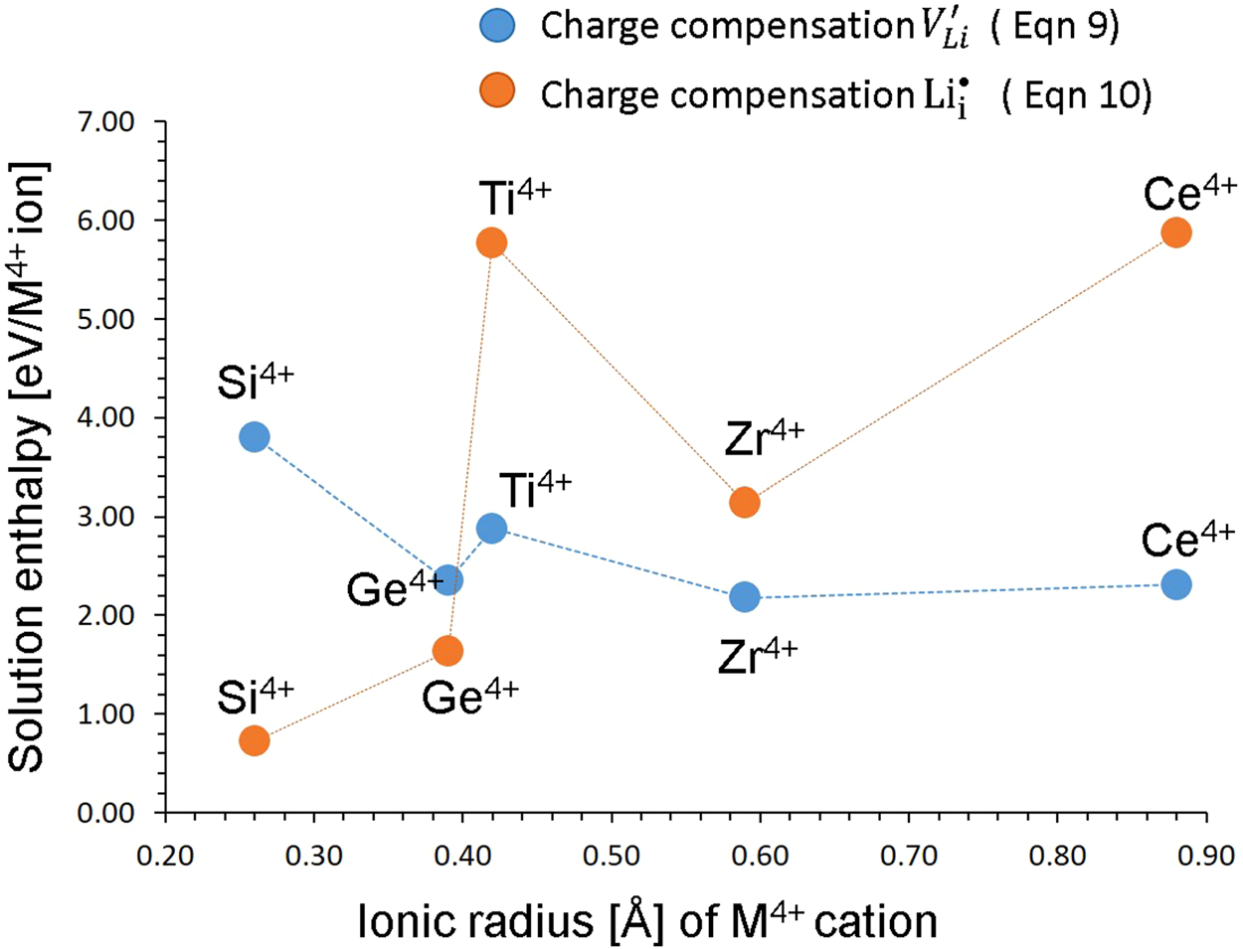
Enthalpy of solution of *RO*_2_ (*R = *Si, Ge, Ti, Zr and Ce) with respect to the R^4+^ ionic radius in Li_3_V_2_(PO_4_)_3_.

Figure 5 reports the solution energies of *RO*_2_ and it can be observed that ZrO_2_ has the lowest solution energy of 2.18 eV. Solution energies of CeO_2_ and GeO_2_ are 2.31 eV and 2.35 eV respectively meaning that Ce and Ge are also promising candidate dopants. As these solution energies are higher compared to the Li Frenkel process, the solution of ZrO_2_, GeO_2_ and CeO_2_ during synthesis should be examined experimentally as they can increase the Li vacancy concentration (via relation (9)).

Incorporation of additional lithium into the as-prepared material will enhance the capacity and further increase the applicability of Li_3_V_2_(PO_4_)_3_ as a viable cathode material for rechargeable sodium batteries. A defect engineering way to increase the amount of lithium is by doping tetravalent cations on P site through creating Li interstitials. The efficacy of the approach has been previously demonstrated experimentally and theoretically in Li battery cathode materials^18^. In a theoretical study of polymorphs of Li_2_MnSiO_4_, it was suggested that Al doping on the Si site is a possible way of introducing addition Li interstitial in Li_2_MnSiO_4_^40^. Here we considered the solution of *RO*_2_(*R* = Si, Ge, Ti, Zr and Ce) via the following process (in Kröger-Vink notation):

10 $$2{{\rm{RO}}}_{2}+2{{\rm{P}}}_{{\rm{P}}}^{{\rm{X}}}+{{\rm{Li}}}_{2}{\rm{O}}\,\to 2{{\rm{R}}{\prime} }_{{\rm{P}}}+2{{\rm{Li}}}_{{\rm{i}}}^{\bullet }+{{\rm{P}}}_{2}{{\rm{O}}}_{5}$$

Figure 5 reports the solution energies of RO_2_ and it can be observed that the most favorable dopant solution energy is found for Si^4+^. This suggests that a possible synthesis-doping strategy of introducing additional lithium into Li_3_V_2_(PO_4_)_3_, although the exact amount of Si incorporation cannot be predicted. The second most favorable dopant is Ge^4+^. The solution energy increases further with the dopant size.

Concentration of Li ions will be dominated by tetravalent doping via two processes as shown in equations 9 and 10. The formation Li interstitials will be favored by Si and Ge dopants (eqn 10) on the P site whereas Ti, Zr and Ce on the V site will favor the formation of Li vacancies (eqn 9). Solution of these tetravalent dopants will create the corresponding Li defects.

### Trivalent doping

A wide range of isovalent substitutions on V sites were considered. The dopant incorporation mechanism does not require the creation of vacancies or interstitials for charge-compensation. Here we considered the solution of R_2_O_3_ (*R* = Al, Ga, Sc, In, Y, Gd and La) via the following process (in Kröger-Vink notation):

11 $${{\rm{R}}}_{2}{{\rm{O}}}_{3}+2{V}_{{\rm{V}}}^{{\rm{X}}}\to 2{{\rm{V}}}_{{\rm{R}}}+{{\rm{V}}}_{2}{{\rm{O}}}_{3}$$

**Figure 6:**
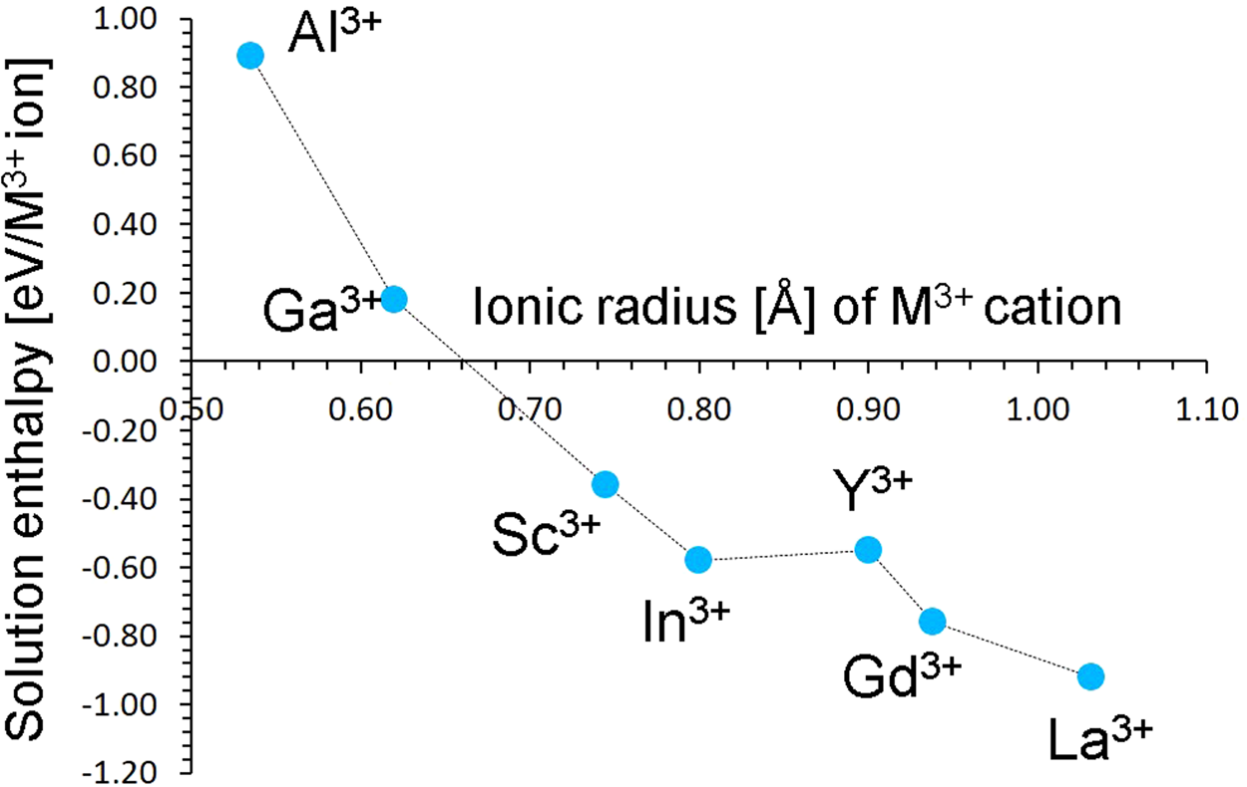
Enthalpy of solution of R_2_O_3_ (*R = *Al, Ga, Sc, In, Y, Gd and La) with respect to the R^3+^ ionic radius in Li_3_V_2_(PO_4_)_3_.

In Fig. 6 the solution energies as a function of the dopant ionic radius are reported. The results reveal that the formation enthalpies for the dopants Sc^3+^, In^3+^, Y^3+^, Gd^3+^ and La^3+^ are exoergic suggesting that they are ideal candidates for substitution at the isovalent V site. The solution enthalpy for La^3+^ is highly negative meaning that it is worth investigating the formation of Li_3_(V,La)_2_(PO_4_)_3_ experimentally and its electrochemical performance.

### Summary

In the present study, the atomistic simulation techniques have been used to provide detailed insights into intrinsic defects, lithium ion mobility and trivalent doping, which are relevant to the general electrochemical behavior of Li_3_V_2_(PO_4_)_3_. The dominant energy defect process is Li Frenkel. The Li-V anti-site defect is calculated to be the second most stable defect process suggesting that there would be small intrinsic concentration of Li on V sites at operating temperatures. The long range Li ion diffusion path with lowest migration energy was calculated to be three dimensional with the migration energy of 0.60 eV. Solution energies of RO_2_ (R = Al, Ga, Sc, In, Y, Gd and La) were calculated to increase Li vacancies and extra Li ions in Li_3_V_2_(PO_4_)_3_. Zr on V site and Si on P site were found to be the efficient stratergies to increase Li vacancies and extra Li ions repectively. Promising candidates for isovalent substitution on V site are Sc, In, Y, Gd and La. The present study aims to inspire further experimental work on doped Li_3_V_2_(PO_4_)_3_.

## Methods

To calculate the energetics for the formation of intrinsic defects and the Li ion diffusion pathways, the classical pair potential method as implemented in the GULP package^43^ was employed. This method is based on the classical Born model description of the ionic crystal lattice. All systems were treated as crystalline solids with interactions between ions consisting of the long-range attractions and short-range repulsive forces representing electron-electron repulsion and van der Waals interactions. The short-range interactions were modelled using Buckingham potentials (refer to Table S1, supplementary information)^30,31,44–48^. Simulation boxes and the corresponding atom positions were relaxed using the Broyden-Fletcher-Goldfarb-Shanno (BFGS) algorithm^49^. The Mott-Littleton method^50^ was used to investigate the lattice relaxation about point defects and the migrating ions. It divides the crystal lattice into two concentric spherical regions, where the ions within the inner spherical region (on the order of >700 ions) immediately surrounding the defect relaxed explicitly. Li ion diffusion was calculated considering two adjacent vacancy sites as initial and final configurations. Seven interstitial Li ions were considered in a direct linear route and they were fixed while all other ions were free to relax. The local maximum energy along this diffusion path is calculated and reported as activation energy of migration. As the present model assumes a full charge ionic model with the calculations corresponding to the dilute limit the defect enthalpies will be overestimated, however, relative energies and trends will be consistent.

## Electronic supplementary material

Defects and dopant properties of Li3V2 (PO4)3
