# Supplementary material for: Defects and dopant properties of Li3V2(PO4)3
Source: Sci Rep. 2019 Jan 23;9:333. doi: 10.1038/s41598-018-36398-w (PMC6344550; doi:10.1038/s41598-018-36398-w)
Supplement: Supplementary file 1 — Defects and dopant properties of Li3V2 (PO4)3 [file 41598_2018_36398_MOESM1_ESM.docx]

**Supporting Information**

**Defects and dopant properties of Li_3_V_2_(PO_4_)_3_**

Navaratnarajah Kuganathan,^1,a^ and Alexander Chroneos^1,2,b)^

^1^Department of Materials, Imperial College London, London, SW7 2AZ, United Kingdom

^2^Faculty of Engineering, Environment and Computing, Coventry University, Priory Street, Coventry CV1 5FB, United Kingdom

Corresponding authors, e-mails: a) n.kuganathan@imperial.ac.uk

b) [alexander.chroneos@imperial.ac.uk](mailto:alexander.chroneos@imperial.ac.uk)

**Table S1**. Interatomic potential parameters used in the atomistic simulations of Li_3_V_2_(PO_4_)_3_.

Two-body [Φ*_ij_* (*r_ij_*) = *A_ij_* exp (− *r_ij_* /*ρ_ij_*) − *C_ij_ / r_ij_*^6^]

| Interaction | *A* (eV) | *ρ* (Å) | *C* (eV·Å^6^) | Y (e) | K (eV·Å^-2^) |
| --- | --- | --- | --- | --- | --- |
| Li^+^–O^2−[1]^ | 632.1018 | 0.2906 | 0.00 | 1.000 | 99999 |
| V^3+^–O^2−[2]^ | 1410.82 | 0.3117 | 0.00 | 2.040 | 196.3 |
| P^5+^–O^2–[2]^ | 897.2648 | 0.3577 | 0.00 | 5.000 | 99999 |
| O^2−^–O^2−[2]^ | 22764.30 | 0.1490 | 27.88 | –2.860 | 74.92 |
| Al^3+^ - O^2−[3]^ | 1114.9 | 0.3118 | 0.000 | 3.000 | 99999 |
| Sc^3+^ - O^2−[3]^ | 1299.4 | 0.3312 | 0.000 | 3.000 | 99999 |
| In^3+^ - O^2−[4]^ | 1495.65 | 0.3327 | 4.33 | 3.000 | 99999 |
| Y^3+^ - O^2−[3]^ | 1345.1 | 0.3491 | 0.00 | 3.000 | 99999 |
| Gd^3+^ - O^2−[5]^ | 1885.75 | 0.3399 | 20.34 | 3.000 | 99999 |
| La^3+^ - O^2−[6]^ | 1545.21 | 0.3590 | 0.00 | ‒0.25 | 145.0 |
| Ga^3+^ - O^2−[3]^ | 2901.12 | 0.2742 | 0.000 | 3.000 | 99999 |
| Si^4+^ - O^2−[7]^ | 1283.91 | 0.32052 | 10.66 | 4.000 | 99999 |
| Ge^4+^ - O^2−[7]^ | 1497.3996 | 0.325646 | 16.00 | 4.000 | 99999 |
| Ti^4+^ - O^2−[7]^ | 5111.700 | 0.2625 | 0.00 | ‒0.10 | 314.0 |
| Zr^4+^ - O^2−[7]^ | 985.869 | 0.3760 | 0.00 | 1.35 | 169.617 |
| Ce^4+^ - O^2−[7]^ | 1986.83 | 0.3511 | 20.40 | 7.70 | 291.75 |

**Table S2.** Energetics of intrinsic defect process in Li_3_V_2_(PO_4_)_3_.

| Defect process/equation | Reaction energy/eV | Reaction energy per defect/eV |
| --- | --- | --- |
| Li Frenkel /1 | 0.90 | 0.45 |
| O Frenkel /2 | 6.00 | 3.00 |
| V Frenkel /3 | 5.94 | 2.97 |
| P Frenkel /4 | 20.08 | 10.04 |
| Schottky /5 | 69.30 | 3.47 |
| Li_2_O Schottky/6 | 6.14 | 3.07 |
| Li/V antisite (isolated) /7 | 3.94 | 1.97 |
| Li/V antisite (cluster) /8 | 1.82 | 0.91 |

**References**

1 Fisher, C. A. J., Hart Prieto, V. M. & Islam, M. S. Lithium Battery Materials LiMPO_4_ (M = Mn, Fe, Co, and Ni): Insights into Defect Association, Transport Mechanisms, and Doping Behavior. *Chem. Mater.* **20**, 5907-5915 (2008).

2 Kuganathan, N., Ganeshalingam, S. & Chroneos, A. Defects, Dopants and Lithium Mobility in Li_9_V_3_(P_2_O_7_)_3_(PO_4_)_2_. *Sci. Rep* **8**, 8140 (2018).

3 Lewis, G. V. & Catlow, C. R. A. Potential models for ionic oxides. *J. Phys C: Solid State Phys.* **18**, 1149 (1985).

4 McCoy, M. A., Grimes, R. W. & Lee, W. E. Planar intergrowth structures in the ZnO-In_2_O_3_ system. ‎*Philos. Mag A* **76**, 1187-1201 (1997).

5 Busker, G., Chroneos, A., Grimes, R. W. & Chen, I.-W. Solution mechanisms for dopant oxides in yttria. *J. Am. Ceram. Soc.* 82, 1553-1559 (1999).

6 Minervini, L., Zacate, M. O. & Grimes, R. W. Defect cluster formation in M_2_O_3_-doped CeO_2_. *Solid State Ionics* **116**, 339-349 (1999).

7 Kuganathan, N., Iyngaran, P. & Chroneos, A. Lithium diffusion in L_i5_FeO_4_. *Sci. Rep* **8**, 5832 (2018).
